# Infections and systemic inflammation are associated with lower plasma concentration of insulin-like growth factor I among Malawian children

**DOI:** 10.1093/ajcn/nqaa327

**Published:** 2020-12-31

**Authors:** Kenneth Maleta, Yue-Mei Fan, Juho Luoma, Ulla Ashorn, Jaden Bendabenda, Kathryn G Dewey, Heikki Hyöty, Mikael Knip, Emma Kortekangas, Kirsi-Maarit Lehto, Andrew Matchado, Minyanga Nkhoma, Noora Nurminen, Seppo Parkkila, Sami Purmonen, Riitta Veijola, Sami Oikarinen, Per Ashorn

**Affiliations:** School of Public Health and Family Medicine, College of Medicine, University of Malawi, Blantyre, Malawi; Center for Child Health Research, Faculty of Medicine and Health Technology, Tampere University, Tampere, Finland; Center for Child Health Research, Faculty of Medicine and Health Technology, Tampere University, Tampere, Finland; Center for Child Health Research, Faculty of Medicine and Health Technology, Tampere University, Tampere, Finland; School of Public Health and Family Medicine, College of Medicine, University of Malawi, Blantyre, Malawi; Institute for Global Nutrition and Department of Nutrition, University of California, Davis, CA, USA; Department of Virology, Faculty of Medicine and Health Technology, Tampere University, Tampere, Finland; Fimlab Ltd, Tampere University Hospital, Tampere, Finland; Center for Child Health Research, Faculty of Medicine and Health Technology, Tampere University, Tampere, Finland; Paediatric Research Center, Children's Hospital, University of Helsinki and Helsinki University Hospital, Helsinki, Finland; Research Programs for Clinical and Molecular Metabolism, Faculty of Medicine, University of Helsinki, Helsinki, Finland; Folkhälsan Research Centre, Helsinki, Finland; Center for Child Health Research, Faculty of Medicine and Health Technology, Tampere University, Tampere, Finland; Center for Child Health Research, Faculty of Medicine and Health Technology, Tampere University, Tampere, Finland; School of Public Health and Family Medicine, College of Medicine, University of Malawi, Blantyre, Malawi; Center for Child Health Research, Faculty of Medicine and Health Technology, Tampere University, Tampere, Finland; Department of Virology, Faculty of Medicine and Health Technology, Tampere University, Tampere, Finland; Fimlab Ltd, Tampere University Hospital, Tampere, Finland; Clinical Medicine, Faculty of Medicine and Health Technology, Tampere University, Tampere, Finland; Clinical Medicine, Faculty of Medicine and Health Technology, Tampere University, Tampere, Finland; Department of Paediatrics, PEDEGO Research Unit, Medical Research Centre, Oulu University Hospital and University of Oulu, Oulu, Finland; Department of Virology, Faculty of Medicine and Health Technology, Tampere University, Tampere, Finland; Center for Child Health Research, Faculty of Medicine and Health Technology, Tampere University, Tampere, Finland; Department of Paediatrics, Tampere University Hospital, Tampere, Finland

**Keywords:** childhood growth faltering, stunting, infection, hormonal regulation, pathway analysis, systemic inflammation

## Abstract

**Background:**

Insulin-like growth factor I (IGF-I) is the most important hormonal promoter of linear growth in infants and young children.

**Objectives:**

The objectives of this study were to compare plasma IGF-I concentration in a low- compared with a high-income country and characterize biological pathways leading to reduced IGF-I concentration in children in a low-income setting.

**Methods:**

We analyzed plasma IGF-I concentration from 716 Malawian and 80 Finnish children at 6–36 mo of age. In the Malawian children, we studied the association between IGF-I concentration and their environmental exposures; nutritional status; systemic and intestinal inflammation; malaria parasitemia and viral, bacterial, and parasitic enteric infections; as well as growth at 18 mo of age. We then conducted a pathway analysis to identify direct and indirect associations between these predictors and IGF-I concentration.

**Results:**

The mean IGF-I concentrations were similar in Malawi and Finland among 6-mo-old infants. At age 18 mo, the mean ± SD concentration was almost double among the Finns compared with the Malawians [24.2 ± 11.3 compared with 12.5 ± 7.7 ng/mL, age- and sex-adjusted difference in mean (95% CI): 11.8 (9.9, 13.7) ng/mL; *P* < 0.01]. Among 18-mo-old Malawians, plasma IGF-I concentration was inversely associated with systemic inflammation, malaria parasitemia, and intestinal *Shigella, Campylobacter*, and enterovirus infection and positively associated with the children's weight-for-length *z* score (WLZ), female sex, maternal height, mother's education, and dry season. Seasonally, mean plasma IGF-I concentration was highest in June and July and lowest in December and January, coinciding with changes in children's length gain and preceded by ∼2 mo by the changes in their WLZ.

**Conclusions:**

The mean plasma IGF-I concentrations are similar in Malawi and Finland among 6-mo-old infants. Thereafter, mean concentrations rise markedly in Finland but not in Malawi. Systemic inflammation and clinically nonapparent infections are strongly associated with lower plasma IGF-I concentrations in Malawi through direct and indirect pathways.

## Introduction

Stunting—that is, having a low length or height for age—is estimated to affect 149 million (21.9%) children <5 y of age worldwide (1). The condition is associated with increased mortality and morbidity, poor cognitive development, low educational attainment, reduced lifetime earnings, and increased risk of chronic diseases in adulthood (2, 3). Because of its high prevalence and the associated deleterious outcomes, stunting among children is now considered a major health threat and its reduction has become a global development priority (4, 5).

Although the global prevalence of stunting has decreased in recent years, the changes are modest and insufficient to meet the global target to reduce by 40% the number of stunted children <5 y of age by 2025 (1, 6). A major impediment for faster progress may be inadequate understanding of the exact biological processes that lead to stunting. According to a widely accepted causation framework, growth restriction is related to undernutrition, frequent illnesses, and other adverse environmental exposures (e.g., poor water source and sanitation) (7). Currently, however, there is little information on the relative importance of these factors and the biological pathways by which they restrict elongation of long bones, which is a hormonally regulated process that results in linear growth in children (8).

In infants and young children, the most important hormonal promoter of linear growth is insulin-like growth factor I (IGF-I), which is produced in liver and other tissues upon stimulation by growth hormone and thyroid hormone (8). Most IGF-I is secreted into the circulation, and it exerts its function by binding to specific receptors in the cells of epiphyseal growth plates and other peripheral tissues. Its synthesis is reduced by malnutrition (9) and systemic inflammation (10), and its plasma concentration shows little diurnal variation but responds rapidly to external exposures such as acute illness (11). Because of this responsiveness, short-term triggers of growth restriction may be better identified by measuring plasma IGF-I concentration than actual increment in length or height, which is typically measured over a minimum period of 3 mo.

In this study, we aimed to characterize biological pathways associated with plasma IGF-I concentration in children in low-income settings by describing the distribution of plasma IGF-I concentrations at 6, 18, and 30 mo of age among a group of children in Malawi and comparing the values to those of a well-growing child population in a high-income country. Furthermore, we aimed to develop a pathway map to illustrate how undernutrition, viral, bacterial, and parasitic infections, systemic and intestinal inflammation, and other maternal, child, and environmental variables are associated with plasma IGF-I concentration, and eventually child's length or height gain, in the Malawian setting.

## Methods

### Study design and concept map

This was a secondary analysis of data and biological samples (plasma and stool) that were prospectively collected as part of a dietary intervention trial in Malawi [the International Lipid-Based Nutrient Supplements Project DYAD–Malawi (iLiNS-DYAD-M)] and from an observational birth cohort study in Finland [Type 1 Diabetes Prediction and Prevention study (DIPP)]. From both cohorts, we analyzed the plasma IGF-I concentration at 6, 18, and 24–36 mo of age in the participants to describe its distribution in apparently healthy children representing different average growth patterns. We then used 2 different statistical approaches to identify direct and indirect predictors of plasma IGF-I concentration at 18 mo—an age when other hormones such as insulin or sex steroids play a minor role in driving child growth (12). The pathway analysis was performed only in the Malawian children, among whom growth restriction was common.

### Study participants

From Malawi, we included children who had participated in the iLiNS-DYAD-M dietary intervention trial and contributed any anthropometric, laboratory, or environmental exposure data to the analysis. The full details of this trial have been described elsewhere (clinicaltrials.gov identifier NCT01239693) (13, 14).

In brief, iLiNS-DYAD-M was a randomized, outcome assessor-blinded, 3-arm intervention trial that took place in rural Malawi from February 2011 to April 2015. The primary aim of the trial was to study the impact of small-quantity lipid-based nutrient supplements (SQ-LNS) on maternal and child outcomes, especially birth size and child's growth.

Participants in the iLiNS-DYAD-M trial were pregnant women who were enrolled before 20 weeks of gestation and their offspring. In the first control group, women were provided with iron and folic acid supplements during pregnancy. In the second control group, women were provided with multiple micronutrient supplements during pregnancy and the first 6 mo of lactation. In the intervention group, women were provided with SQ-LNS during pregnancy and the first 6 mo of lactation and children were provided with SQ-LNS from 6 to 18 mo of age. The children were followed-up until 30 mo of age, with regular anthropometric assessment and biological sample collection at 6, 18, and 30 mo of age. At the time of biological sample collection, all children were apparently healthy—that is, their caretakers did not report any illness symptoms for the children.

The Finnish sample was derived from the DIPP study (clinicaltrials.gov identifier NCT03269084) – a population-based long-term clinical follow-up study established since 1994 in 3 university hospitals in Finland. The aim of the Finnish DIPP study is to understand the pathogenesis of type 1 diabetes, predict the disease, and find preventive treatments. Recruitment is based on screening of HLA-DR-DQ genotypes from cord blood sample. Children who carry HLA genotypes associated with increased risk to develop type 1 diabetes are invited to follow-up and monitored for the development of islet autoimmunity. The subjects of the current analysis participated in regular follow-up visits from early infancy to 15 y of age or until type 1 diabetes was diagnosed, with clinical examination and biological sample collection at the ages of 3, 6, 9, 12, 18, and 24 mo and thereafter once a year (15). The participants selected for this analysis were from the clinical DIPP center at Tampere University Hospital, Finland.

Ethical approval was obtained from the College of Medicine Research Ethics Committee, University of Malawi, and the Ethics Committee of Pirkanmaa Hospital District. Only participants whose caregivers gave an informed consent were enrolled in the study.

### Biological specimen collection and processing

Clinic nurses collected the nonfasting Malawian blood samples from the antecubital vein. A trained lab technician separated plasma into storage vials. Stool samples were collected by mothers from participating children in their homes. If a child had diarrhea, no stool sample was collected, and the visit was postponed by 2 wk. Plasma and stool samples were later shipped to Tampere University in Finland and Washington University in St. Louis, MO, on dry ice for analysis. For the current study, we included data from all participants at the ages of 6, 18, and 30 mo with any postnatal anthropometric measurements and any plasma or stool samples.

In the DIPP study, study nurses collected blood samples from the antecubital vein. Blood samples were taken using CPT tubes (BD Biosciences), and plasma was separated according to the manufacturer's instructions into storage vials and stored at –80°C until analysis.

### Analyses of plasma IGF-I concentration

Carrier-protein–free IGF-I concentration was analyzed from stored plasma samples using the commercial MILLIPLEX^®^ MAP HIGF-I, II Magnetic Bead Panel Kit (catalog no. HIGFMAG-52K; EMD Millipore), according to the manufacturer's instructions. Properly diluted plasma samples were incubated with the antibody-coupled microspheres and then with biotinylated detection antibody before the addition of streptavidin–phycoerythrin. The captured bead complexes were measured with the Bio-Plex^®^ 200 system (Bio-Rad Laboratories). The detection range of the assay was 0.12–88.2 ng/mL.

### Other laboratory analyses

Plasma C-reactive protein (CRP) and α1-acid glycoprotein (AGP) were analyzed on a Roche Cobas 6000 analyzer (Roche Diagnostics). Plasma cytokines—IL-1β, IL-6, IL-10, and TNF-α—were measured using the commercial MILLIPLEX^®^ MAP kit (EMD Millipore). Malaria was diagnosed on-site from finger-prick blood samples using the rapid diagnostic test Clearview Malaria Combo (British Biocell International). Maternal HIV infection at study enrollment was tested with a whole-blood antibody rapid test (Alere Determine HIV-1/2; Alere Medical); for children, no HIV tests were done.

Stool samples were assayed using commercially available ELISA kits for calprotectin (Hycult Biotech). *Campylobacter* (16), *Shigella* (17), *Cryptosporidium* spp., *Giardia* (18), enterovirus, rhinovirus, parechovirus, norovirus, and rotavirus infections were detected using an in-house real-time PCR assay that has been shown to be specific and sensitive for detecting these microbes, including the rhinovirus, from stool samples (19, 20).

Microbiota data were obtained from the stool samples using previously described DNA extraction and high-throughput 16S sequencing methods (21, 22). The microbiota-for-age *z* score (MAZ) as a measure of microbiota maturity was obtained by comparing the microbiota ages of participants to the median microbiota age of same-aged children in the reference group generated with a random forests (RF)–derived model, as described previously (21, 22).

### Anthropometric assessment

Trained anthropometrists measured the mothers’ and children's weight and length/height in triplicate, as described previously (13, 14). We calculated age- and sex-standardized anthropometric indices—length-for-age *z* score (LAZ), weight-for-age *z* score (WAZ), and weight-for-length *z* score (WLZ)—using the WHO Child Growth Standards (23). No outlier data points were removed from the data before analysis.

### Morbidity and other data collection

Data collectors visited the children's homes weekly to collect child's morbidity information, which was recorded using a picture calendar by a caregiver. Morbidity variables included fever, diarrhea, and respiratory symptoms recorded in the previous 7 d. No antibiotic exposure data were collected.

Sociodemographic information was collected through personal interviews with mothers. A household assets score was created using principal component analysis based on information on building materials of the house, electricity, and cooking fuel.

### Pathway analysis

We based our pathway analysis on a concept map (**Supplemental Figure 1**), according to which plasma IGF-I concentrations would be directly predicted by the child's current nutritional status (WLZ) and systemic inflammation marker (AGP) (10, 11, 24). In addition, we hypothesized that maternal height and nutritional status [BMI (in kg/m^2^)] would be associated with the child's plasma IGF-I concentration through a parental genetic component shared with the child and nutrients or growth factors given to the child through breast milk (25–28). Moreover, we expected a seasonal effect on IGF-I expression as documented in several animal species (29–31).

The presumed indirect predictors covered mothers’ education; children's fecal calprotectin concentration as a marker of intestinal inflammation; malaria; bacterial (*Campylobacter* and *Shigella*), parasitic (*Giardia* and *Cryptosporidium*), and viral (enterovirus, rhinovirus, parechovirus, norovirus, and rotavirus) infections; intestinal microbiota composition (MAZ); children's clinical morbidity (fever, cough, or diarrhea); and socioeconomic and environmental exposures (household assets, water source, and sanitation). Factors that could be part of the etiological pathway but could not be identified as predictors with the collected data are marked with a dashed line in Supplemental Figure 1.

### Statistical analysis

We compared proportions, means, and SDs between groups by using Student's *t* test for continuous variables and Fisher's exact test for proportions. For selected analyses, we summarized systemic inflammation markers (CRP, AGP, IL-1β, IL-6, IL-10, and TNF-α) into a single principal component and treated the principal component score as a continuous variable. We divided the AGP, CRP, fecal calprotectin, and the principal component score variables into quintiles, and we tested the dose–response of child's plasma IGF-I concentration by quintiles of the predictor using the extended Wilcoxon's rank-sum test (32).

The monthly change in *z* scores of LAZ and WLZ was calculated using linear models to obtain an estimate. The estimates were standardized for the relative monthly changes in *z* scores, and seasonal fluctuation during the year was illustrated by using locally weighted scatterplot smoothing.

We identified the predictors of plasma IGF-I concentration by examining the relations between the dependent and independent variables. For dichotomous predictor variables, we calculated the mean plasma IGF-I concentration per group and tested the difference between groups. For continuous predictors, we assessed linear relations graphically with scatterplots and used linear models to assess the strength of the associations.

In order to meet the assumption of normality in the models, we assessed the normality of each variable and applied natural logarithmic transformation in cases with extreme skewness. For principal component scores, the variables were also centered and scaled. Normalization procedures were not applied to child's plasma IGF-I concentration because central limit theorem dictates that parametric analysis of means is valid and robust despite the shape of the outcome variable's distribution, if the sample size is large (33). The continuous variables were standardized in the generalized structural equation model (GSEM) for easier interpretation of the associations.

We analyzed the causal relations between direct and indirect predictors and plasma IGF-I concentration with the structural equation model (SEM) and GSEM. GSEM was selected as the method of choice for the final pathway model because *1*) it allows for the usage of a variable as both endogenous (outcome) and exogenous (predictor) in the same model and *2*) as opposed to SEM, GSEM allows for usage of generalized linear models. This enabled the use of binary or count variables as outcome variables in the modeling. Missing data were handled by using the full-information maximum-likelihood method in the SEM model and multiple imputation using chained equations in the GSEM model. The set of variables included in the pathway model was determined by a series of generalized linear models.

We conducted sensitivity analysis using an RF machine learning method, which utilized an ensemble of regression models to predict the value of an outcome variable, child's plasma IGF-I concentration. With RF, we tested the robustness of identified predictors in the pathway model by determining the importance of the explanatory variables with the change in mean squared error. Missing values for predictors were imputed into the data using proximity from the RF model. The number of trees used in the model was 1000, and number of variables available for splitting nodes was set to 5.

All statistical analyses were done using either Stata version 15.1 (StataCorp) or R version 3.4.4 software (R Foundation for Statistical Computing).

## Results

Among 790 live-born Malawian infants, 697 (87.5%), 691 (86.7%), and 597 (74.9%) had available anthropometric data at 6, 18, and 30 mo, respectively (**Supplemental Figure 2**). Of the 80 Finnish participants, we had 78, 79, 62, and 63 plasma samples from children at 6, 18, 24, and 36 mo, respectively. At 6 mo of age, the mean ± SD LAZ, WAZ, and WLZ of Malawian infants were –1.26 ± 1.14, –0.57 ± 1.18, and 0.36 ± 1.15, and the corresponding *z* scores for Finnish infants were 0.77 ± 1.00, 0.72 ± 1.04, and 0.49 ± 1.15, respectively (**Table 1**). The proportion of boys was slightly higher in the Finnish than the Malawian sample (54.2% compared with 47.2%), but the difference was not statistically significant (*P* = 0.29) (Table 1). Malawian infants excluded from this study were on average similar to the included ones, except that their mean household asset score was higher and their mothers’ mean BMI was slightly lower (Table 1). The proportion of mothers with a positive HIV test was 11.9%.

**TABLE 1:** Baseline characteristics of the included and excluded Malawian and Finnish children^1^

	Malawi			
Characteristic	Included	Excluded	*P* value	Finland
Participants, *n*	716	80		80
Age, y	0.5 ± 0.08	—		0.5 ± 0.03
Proportion of boys, %	47.2	52.0	0.37	54.2
LAZ	–1.26 ± 1.14	—		0.77 ± 1.0
WAZ	–0.57 ± 1.18	—		0.72 ± 1.04
WLZ	0.36 ± 1.15	—		0.49 ± 1.15
Unsafe water source,^2^ %	9.0	6.7	0.67	
Unsafe sanitary facilities,^3^ %	90.2	93.4	0.53	
Household asset *z* score	–0.05 ± 0.97	0.43 ± 1.16	<0.001	
Maternal age at enrollment, y	25 ± 6	24 ± 7	0.61	
Maternal height, cm	156.3 ± 5.7	155.7 ± 5.4	0.68	
Maternal BMI, kg/m^2^	21.6 ± 2.8	22.9 ± 3.1	0.04	
Mother's education, y	3.9 ± 3.5	4.0 ± 3.5	0.76	

1 Values are means ± SDs or percentages unless otherwise indicated. Student's *t* test for continuous variables and Fisher's exact test for proportions. LAZ, length-for-age *z* score; WAZ, weight-for-age *z* score; WLZ, weight-for-length *z* score.

2 Unprotected well, lake, or pond.

3 Regular pit latrine or no latrine.

**FIGURE 1:**
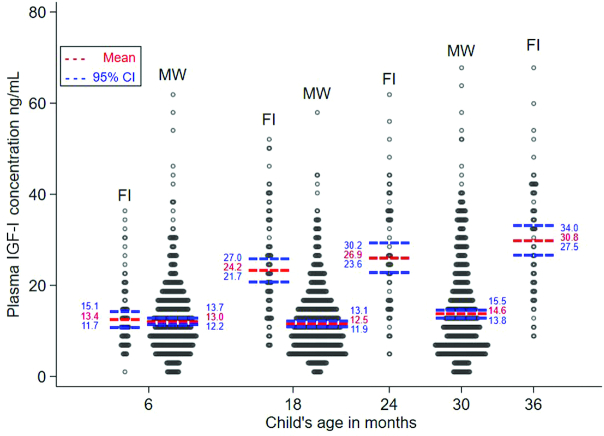
The distribution of plasma IGF-I concentration among Finnish and Malawian infants and children aged 6–36 mo (FI: *n* = 78, 79, 62, and 63 at 6, 18, 24, and 36 mo, respectively; MW: *n* = 520, 606, and 580 at 6, 18, and 30 mo, respectively). At age 18 mo, the age- and sex-adjusted difference in the mean plasma IGF-I concentration between Finnish and Malawian children was 11.8 ng/mL (95% CI: 9.9, 13.7 ng/mL; *P* < 0.01). Student's *t* test was used to detect differences between groups. FI, Finnish; IGF-I, insulin-like growth factor I; MW, Malawian.

In Finland, the mean ± SD plasma IGF-I concentrations were 13.4 ± 7.6, 24.2 ± 11.3, 26.9 ± 13, and 30.8 ± 12.9 ng/mL at 6, 18, 24, and 36 mo of age, respectively. In Malawi, they were 13.0 ± 8.2, 12.5 ± 7.7, and 14.6 ± 10.7 ng/mL at 6, 18, and 30 mo of age, respectively. Accordingly, the mean IGF-I concentration was similar in Finland and Malawi at 6 mo of age, whereas at age 18 mo and older, the mean concentration was higher in Finland than in Malawi [age- and sex-adjusted difference (95% CI) at 18 mo: 11.8 (9.9, 13.7) ng/mL; *P* < 0.01] (**Figure 1**). In relative terms, at 18 mo of age, the Finnish children had a 94% (95% CI: 73, 114%) higher mean plasma IGF-I concentration than the Malawian children.

**FIGURE 2:**
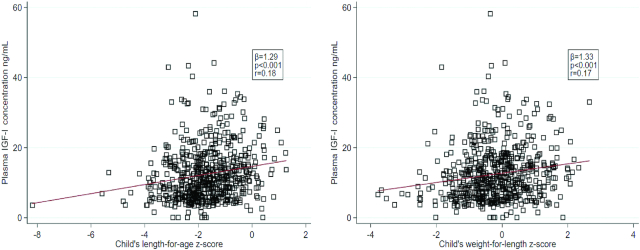
The association between plasma IGF-I concentration and length-for-age and weight-for-length *z* scores at age 18 mo among Malawian children (*n* = 604). Correlation analysis was used to quantify the relation between each pair of variables. IGF-I, insulin-like growth factor I.

There was a weak, albeit significant, association between Malawian children's LAZ and WLZ at 18 mo of age and their concurrent plasma IGF-I concentration (*r* = 0.18, *P* < 0.001 and *r* = 0.17, *P* < 0.001, respectively) (**Figure 2**).

Systemic inflammation was strongly associated with a lower plasma IGF-I concentration, in a dose-dependent manner, with children in the highest inflammation quintile having almost 50% lower mean IGF-I plasma concentration than children in the lowest inflammation quintile (**Table 2**). In contrast, there was no association between the children's plasma IGF-I concentration and their intestinal inflammation, as assessed by stool calprotectin concentration (Table 2).

**TABLE 2:** The association between systemic or intestinal inflammation and plasma IGF-I concentration among Malawian children at 18 mo of age^1^

	Mean plasma IGF-I concentration among children with various levels of inflammation, ng/mL					
Marker of inflammation	1st quintile^2^	2nd quintile	3rd quintile	4th quintile	5th quintile	*P* value^3^
Plasma AGP concentration	15.2	13.0	11.9	9.1	7.3	<0.001
Plasma CRP concentration	14.9	14.0	12.9	11.4	8.9	<0.001
Principal component score for systemic inflammation	15.2	13.4	12.0	10.9	8.9	<0.001
Fecal calprotectin concentration	12.8	12.7	12.3	12.6	11.5	0.66

1 AGP, α1-acid glycoprotein; CRP, C-reactive protein; IGF-I, insulin-like growth factor I.

2 Children in the lowest quintile of the inflammation marker (lowest 20%).

3 *P* value obtained using Cuzick's Wilcoxon-type test for trend.

Children with intestinal *Campylobacter* or *Shigella* infection or blood malaria parasitemia had a significantly lower mean plasma IGF-I concentration compared with their uninfected peers, whereas no similar association was evident for intestinal *Cryptosporidium* or *Giardia* infection (**Table 3**). There was also no association between plasma IGF-I concentration and the detection of noro-, parecho-, or rhinovirus in the stool. Children with intestinal enterovirus or rotavirus infection had a lower mean plasma IGF-I concentration, but the difference was not statistically significant (*P* = 0.051 for enterovirus and *P* = 0.52 for rotavirus) (**Table 4**). No linear relation was observed between the plasma IGF-I concentration and MAZ or children's clinical morbidity (fever, cough, or diarrhea) within the past 7 d (data not shown).

**TABLE 3:** The association between selected bacterial and parasitic infections, malaria, and plasma IGF-I concentration among Malawian children at 18 mo of age^1^

	Plasma IGF-I concentration,^2^ ng/mL			
Detected infection (no. of children with negative/positive test result)	Children with negative test result for infection	Children with positive test result for infection	Difference (95% CI)	*P* value^3^
Intestinal *Campylobacter* (186/405)	13.6 ± 6.7	11.8 ± 9.2	–1.8 (–3.3, –0.3)	0.021^4^
Intestinal *Shigella* (538/51)	12.7 ± 7.7	9.4 ± 5.7	–3.3 (–5.0, –1.6)	<0.001
Intestinal parasite				
Intestinal *Cryptosporidium* (573/15)	12.5 ± 7.5	12.4 ± 13.6	–0.04 (–4.0, 3.9)	0.22^5^
Intestinal *Giardia* (270/318)	12.8 ± 7.8	12.1 ± 7.6	–0.7 (–2.0, 0.6)	0.27
Blood parasite				
Blood malaria parasitemia (524/61)	12.8 ± 7.7	9.9 ± 7.4	–2.9 (–4.9, –0.8)	0.01

1 IGF-I, insulin-like growth factor I.

2 Means ± SDs.

3 *P* values obtained using Student's *t* test.

4 Degrees of freedom for the *t* test obtained from Welch's formula due to unequal variances.

5 Wilcoxon–Mann–Whitney sum of ranks test.

**TABLE 4:** The association between selected viral infections and plasma IGF-I concentration among Malawian children at 18 mo of age^1^

	Plasma IGF-I concentration,^2^ ng/mL			
Detected infection (no. of children with negative/positive test results)	Children with negative test result for infection	Children with positive test result for infection	Difference (95% CI)	*P* value^3^
Enterovirus (88/500)	14.2 ± 9.0	12.2 ± 7.4	–2.0 (–4.0, 0.01)	0.051^4^
Rhinovirus (560/28)	12.4 ± 7.7	13.7 ± 7.6	1.3 (–1.7, 4.2)	0.40
Parechovirus (498/90)	12.5 ± 17.4	12.0 ± 9.1	–0.6 (–2.3, 1.2)	0.51
Norovirus (541/47)	12.5 ± 7.8	11.9 ± 7.0	–0.6 (–2.9, 1.7)	0.63
Rotavirus (584/4)	12.5 ± 8.7	10.4 ± 7.7	–2.1 (–9.7, 5.5)	0.52^5^

1 IGF-I, insulin-like growth factor I.

2 Means ± SDs.

3 *P* values obtained using Student's *t* test.

4 Degrees of freedom for the *t* test obtained from Welch's formula due to unequal variances.

5 Wilcoxon–Mann–Whitney sum of ranks test.

The plasma IGF-I concentration in Malawian children was positively associated with maternal education and sanitation. Other environmental exposures (water source and household assets) and remaining maternal characteristics (height and BMI) were not directly associated with the plasma IGF-I concentration in the children (**Supplemental Table 1**).

**FIGURE 3:**
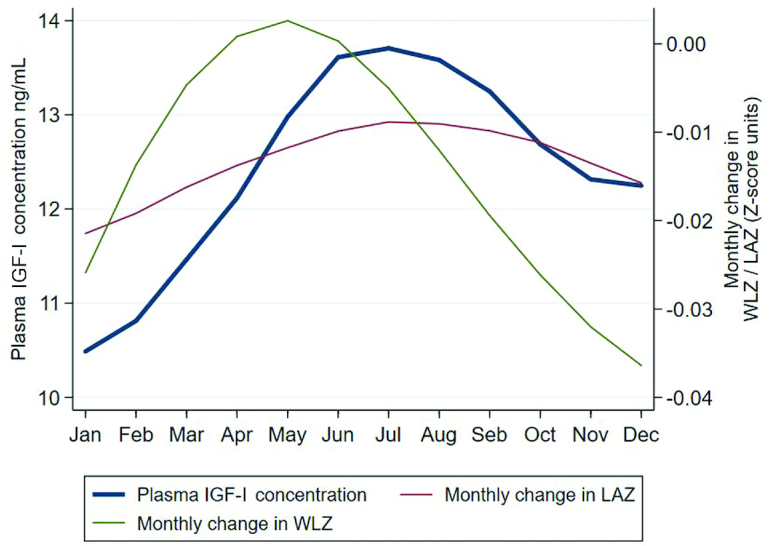
Seasonal variation among Malawian children in plasma IGF-I concentration and standardized monthly change in their WLZ and LAZ at age 18 mo (*n* = 605). The monthly change in WLZ and LAZ was calculated using linear models to obtain an estimate. The estimates were standardized for the relative monthly changes in z scores, and seasonal fluctuation during the year was illustrated by using locally weighted scatterplot smoothing. IGF-I, insulin-like growth factor I; LAZ, length-for-age z score; WLZ, weight-for-length *z* score.

**Figure 3** shows the variation in mean plasma IGF-I concentration and the standardized change in LAZ and WLZ by calendar month among 18-mo-old Malawian children. All seasonal curves followed a similar pattern, but with slightly different schedules. The mean average monthly change in WLZ peaked in May and June, whereas both the plasma IGF-I concentration and the mean average monthly change in LAZ peaked 1–2 mo later, in June–August (Figure 3).

**FIGURE 4:**
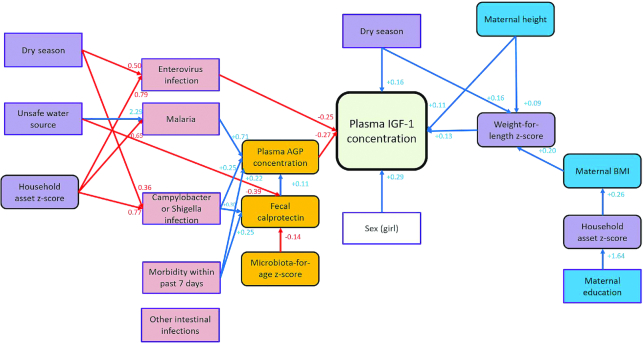
Pathway model for the determinants of plasma IGF-I concentration among Malawian children aged 18 mo. Red lines indicate negative associations, and blue lines indicate positive associations. The hypothesized causal relations between direct and indirect predictors and plasma IGF-I concentration were analyzed with SEM and GSEM. AGP, α1-acid glycoprotein; GSEM, generalized structural equation modeling; IGF-I, insulin-like growth factor I; SEM, structural equation modeling.

**Figure 4** shows results from the GSEM analysis, as a visualization of the direct and indirect predictors of plasma IGF-I concentration among 18-mo-old Malawian children. IGF-I concentration was inversely associated with systemic inflammation and intestinal enterovirus infection and positively associated with the children's WLZ, female sex, maternal height, and dry season. Other variables in the pathway map were associated with the plasma IGF-I concentration indirectly (Figure 4). The indicated model had a good fit (root mean square error of approximation = 0.038, Tucker–Lewis index = 0.82, and comparative fit index = 0.91), and it explained 44% of the variance in plasma IGF-I concentration.

The importance of systemic inflammation, WLZ, and child's sex as predictors of the children's plasma IGF-I concentration was confirmed in an RF model (**Supplemental Figure 3**). Systemic inflammation was the most important variable in predicting plasma IGF-I concentration (improving the accuracy by 18.4%), followed by child sex (10.7%), WLZ (6.3%), malaria infection (4.1%), MAZ (2.3%), and enterovirus infection (1.3%) (Supplemental Figure 3).

## Discussion

We aimed to characterize biological pathways associated with plasma IGF-I concentration in children in a low-income setting by describing their plasma IGF-I concentration and identifying direct and indirect predictors of the IGF-I concentration. The mean plasma IGF-I concentrations were similar in the 2 countries in children aged 6 mo. Thereafter, plasma IGF-I concentrations increased among the Finnish but not the Malawian children, so that at age 18 mo, the mean value in Malawi was only half of that in Finland. Among the Malawians, plasma IGF-I concentration was inversely associated with systemic inflammation and intestinal enterovirus infection and positively associated with the children's WLZ, female sex, maternal height, and dry season. Malaria parasitemia, intestinal *Shigella* and *Campylobacter* infection, and intestinal inflammation were positively associated with systemic inflammation and hence indirectly also with plasma IGF-I concentration, whereas there was no similar association for intestinal *Giardia, Cryptosporidium*, or other viral infections. Seasonally, mean plasma IGF-I concentrations peaked in June and July and showed a nadir in December and January, coinciding with changes in children's length gain and preceded by ∼2 mo by the changes in their WLZ.

Internal validity could have been compromised by errors or differences in laboratory processes, missing data, or the choice of modeling techniques. External validity could have been affected by the choice of variables in the analyses. However, we used similar and standardized biological sample collection, processing, and storage procedures in Malawi and Finland and performed all laboratory assays according to the manufacturer's instructions. Furthermore, the excluded participants and those with missing data had on average similar baseline characteristics compared with participants who provided data, and we used multiple imputations to model the missing values (34). Finally, we included variables that had been considered appropriate in previous analyses (10, 11, 24, 35), and the results were robust to sensitivity analyses with RF modeling. Therefore, we believe that the sample findings are valid and representative of the population from which they were drawn.

According to earlier literature, mean plasma IGF-I concentrations in normally well-growing children are lowest at 6–8 mo of age, rise relatively steadily until puberty, and then fall to lower adult concentrations (36–39). Our findings in the Finnish sample are thus similar to those observed in Sweden (36), Turkey (37), Denmark (38), and Germany (39). Similarly, our findings from Malawi match those from a study in Zimbabwe, documenting low mean plasma IGF-I concentrations at 6 and 18 mo of age (40). These results suggest that the age-dependent rise in plasma IGF-I concentration is prevented or delayed in some low-income settings, leading to growth faltering in late infancy and early childhood. This would fit well with the infancy–childhood–puberty model of growth, according to which the mostly nutrition-dependent early infancy growth is gradually replaced by growth-hormone– and IGF-I–dependent childhood growth, which is eventually superimposed by a sex-steroid–driven growth spurt during puberty (41). The growth hormone–dependent growth is strengthened between 6 and 12 mo of age, and a delay in this phase has been suggested to explain at least part of the linear growth faltering seen in low-income settings (42, 43).

The fact that Malawian infants were on average markedly shorter than the Finnish infants at 6 mo of age, despite a similar mean plasma IGF-I concentration, can partially be explained by a high proportion of preterm births. Because of a shorter fetal period, newborns in the iLiNS-DYAD-M trial had less time to grow and they were shorter and lighter than children in an international reference already at birth (14). In addition, during the fetal period and early infancy, linear growth is promoted by other hormones that we did not study, such as IGF-II, insulin, and thyroid hormones (8). Thus, there may have been a difference in the hormonal drivers of growth before 6 mo of age.

The list of predictors of children's plasma IGF-I that we identified is mostly consistent with previous literature. Weight-for-length/height stimulates growth-hormone and IGF-I production in children (12); cough, fever, and diarrhea are inversely associated with plasma IGF-I concentration; and inflammatory cytokines interfere with IGF-I expression in liver cells (10–12, 44). Girls have been repeatedly shown to have higher mean IGF-I concentration compared with boys, likely because of their genetic constitution (36, 37, 39), and the same probably applies to children of taller mothers. We could not find earlier human studies on the seasonality of plasma IGF-I concentration, but vitamin D has been found to increase IGF-I concentration in adults (45), and the seasonality phenomenon has been documented and associated with light exposure in many animal species (29–31). Additional exposures that were not included in our models but are likely to explain part of the remaining variability in children's plasma IGF-I concentration include their genetic constitution, liver function, activity of other growth-related hormones, nutritional exposures not reflected in concurrent WLZ, and children's HIV status (25–27).

One striking finding in our analysis was the consistent associations between microbial detection in blood or stools and reduced IGF-I concentration among children who did not have apparent disease symptoms at the time of the biospecimen collection. The impact of acute illness on linear growth is well documented (46–49), and this impact has often been attributed to anorexia, reduced nutrient intake, nutrient loss, increased catabolism, and nutrient sequestration (50, 51). A study in rural Zimbabwe suggested that children with acute infections have reduced plasma IGF-I concentration (11), and similar results have been reported in relation to many other symptomatic infections, such as bacterial sepsis (52), leprosy (53), and hepatitis C (54). Thus, infection- and inflammation-related IGF-I downregulation seems to be another important mechanism that links infection, disease, and growth restriction in low-income settings. Our results and those of others suggest that such downregulation also takes place during many clinically nonapparent infections (44, 55). The direct association between enteroviral carriage and lower plasma IGF-I concentration could be a spurious finding. However, it might also be a true causal association because enteroviral infection has been associated with a marked downregulation of IGF-II expression in a cell-culture condition (56).

We could not find any other study that examined concurrent seasonal patterns in plasma IGF-I concentration, change in length for age, and change in weight for length among children. However, several previous studies have described a seasonal pattern of ponderal and linear growth in low-income settings, with peak annual weight gains typically following harvest season and preceding peak height gains by some months (57, 58). Similarly, in the recovery from severe acute malnutrition, children typically first gain weight before they start gaining length/height (59). These findings and ours are compatible with a theory that increased food security and intake promotes fat deposition in growing children, resulting in leptin synthesis and somewhat later enhanced growth hormone and IGF-I secretion and increased length or height gain. Additional seasonal effects could be mediated by variation in infection prevalence and inflammation, and possibly light exposure.

Two limitations of our study are the small sample size and limited data availability for the Finnish cohort and lack of direct evidence between plasma IGF-I concentration of children's linear growth in the Malawian sample. Therefore, we could not complete full pathway analyses for both cohorts. The sample sizes were, however, sufficient to demonstrate marked differences in the mean IGF-I concentration between the populations. And because IGF-I is known to be the main driver of growth among 18-mo-old children (8), the variance in its concentration is likely to explain a significant proportion of the observed growth difference between the 2 populations. Furthermore, plasma IGF-I concentration that swiftly responds to external stimuli is likely a better outcome variable than linear growth for studies identifying factors that restrict child growth. This is because many of the exposures (e.g., infection) are temporary and hence exposure status at 1 point is not predictive for the subsequent 3–6 mo, which is typically the shortest interval for length measurement.

In summary, we have shown that IGF-I concentrations are considerably lower among 18- to 36-mo-old children in Malawi than in Finland, that seasonal variation in children's growth parallels very closely that of plasma IGF-I concentration, and that systemic inflammation and clinically nonapparent infections are common and strongly associated with reduced plasma IGF-I concentrations in Malawi. Based on these findings and the literature that we reviewed, we argue that broad and effective infection prevention and management programs are essential for successful promotion of healthy child growth in low-income settings.

## Supplementary Material

nqaa327_Supplemental_File

## Data Availability

Data described in the manuscript, code book, and analytic code will be made available upon request pending approval by the authors.

## Notes

This study was funded by the Finnish Funding Agency for Technology and Innovation; the Bill & Melinda Gates Foundation through the University of California, Davis; the Office of Health, Infectious Diseases, and Nutrition, Bureau for Global Health, US Agency for International Development under terms of Cooperative Agreement AID-OAA-A-12-00005 through the Food and Nutrition Technical Assistance III Project, managed by FHI 3603; the Foundation for Paediatric Research in Finland; and the competitive research funding of the Tampere University Hospital (9AA004). The funders had no role in study design, data collection and analysis, decision to publish, or preparation of the manuscript.

Supplemental Figures 1–3 and Supplemental Table 1 are available from the “Supplementary data” link in the online posting of the article and from the same link in the online table of contents at https://academic.oup.com/ajcn/.

Abbreviations used: AGP, α1-acid glycoprotein; CRP, C-reactive protein; DIPP, Type 1 Diabetes Prediction and Prevention study; GSEM, generalized structural equation model; IGF-I, insulin-like growth factor I; iLiNS-DYAD-M, International Lipid-Based Nutrient Supplements Project DYAD–Malawi; LAZ, length-for-age *z* score; MAZ, microbiota-for-age *z* score; RF, random forests: SEM, structural equation model; SQ-LNS, small-quantity lipid-based nutrient supplement; WAZ, weight-for-age *z* score; WLZ, weight-for-length *z* score.

## References

[1] UNICEF, WHO, International Bank for Reconstruction and Development/World Bank Levels and trends in child malnutrition: key findings of the 2019 edition of the Joint Child Malnutrition Estimates. Geneva (Switzerland): WHO; 2019.

[2] Hoddinott J , Behrman JR, Maluccio JA, Melgar P, Quisumbing AR, Ramirez-Zea M, Stein AD, Yount KM, Martorell R Adult consequences of growth failure in early childhood. Am J Clin Nutr. 2013;98:1170–8. 24004889 10.3945/ajcn.113.064584 PMC3798075

[3] Victora CG. Nutrition in early life: a global priority. Lancet North Am Ed. 2009;374:1123–5. 10.1016/S0140-6736(09)61725-6 19801082

[4] WHO Resolution WHA65.6: Comprehensive implementation plan on maternal, infant and young child nutrition. In: Sixty-fifth World Health Assembly, Geneva, 21–26 May. Resolutions and decisions, annexes [Internet] Geneva (Switzerland): WHO; 2012; [cited 15 December, 2019] Available from: https://apps.who.int/gb/ebwha/pdf_files/WHA65-REC1/A65_REC1-en.pdf and https://apps.who.int/gb/ebwha/pdf_files/WHA65/A65_11Corr1-en.pdf.

[5] UN General Assembly Report of the Open Working Group of the General Assembly on Sustainable Development Goals. [Internet] 2014; [cited 16 December, 2019] Available from: https://www.un.org/ga/search/view_doc.asp?symbol=A/68/970&Lang=E.

[6] de Onis M , Dewey KG, Borghi E, Onyango AW, Blossner M, Daelmans B, Piwoz E, Branca F The World Health Organization's global target for reducing childhood stunting by 2025: rationale and proposed actions. Matern Child Nutr. 2013;9(Suppl 2):6–26. 10.1111/mcn.12075 PMC6860845 24074315

[7] Millward DJ Nutrition, infection and stunting: the roles of deficiencies of individual nutrients and foods, and of inflammation, as determinants of reduced linear growth of children. Nutr Res Rev. 2017;30:50–72. 28112064 10.1017/S0954422416000238

[8] Rosenbloom AL Physiology of growth. Ann Nestle. 2007;65:97–108.

[9] Goldstein S , Harp JB, Phillips LS Nutrition and somatomedin. XXII: Molecular regulation of insulin-like growth factor-I during fasting and refeeding in rats. J Mol Endocrinol. 1991;6:33–43. 2015055 10.1677/jme.0.0060033

[10] Walters TD , Griffiths AM Mechanisms of growth impairment in pediatric Crohn's disease. Nat Rev Gastroenterol Hepatol. 2009;6:513–23. 19713986 10.1038/nrgastro.2009.124

[11] Jones AD , Rukobo S, Chasekwa B, Mutasa K, Ntozini R, Mbuya MN, Stoltzfus RJ, Humphrey JH, Prendergast AJ Acute illness is associated with suppression of the growth hormone axis in Zimbabwean infants. Am J Trop Med Hyg. 2015;92:463–70. 25535308 10.4269/ajtmh.14-0448 PMC4347356

[12] Benyi E , Savendahl L The physiology of childhood growth: hormonal regulation. Horm Res Paediatr. 2017;88:6–14. 28437784 10.1159/000471876

[13] Ashorn P , Alho L, Ashorn U, Cheung YB, Dewey KG, Gondwe A, Harjunmaa U, Lartey A, Phiri N, Phiri TE et al. Supplementation of maternal diets during pregnancy and for 6 months postpartum and infant diets thereafter with small-quantity lipid-based nutrient supplements does not promote child growth by 18 months of age in rural Malawi: a randomized controlled trial. J Nutr. 2015;145:1345–53. 25926413 10.3945/jn.114.207225

[14] Ashorn P , Alho L, Ashorn U, Cheung YB, Dewey KG, Harjunmaa U, Lartey A, Nkhoma M, Phiri N, Phuka J et al. The impact of lipid-based nutrient supplement provision to pregnant women on newborn size in rural Malawi: a randomized controlled trial. Am J Clin Nutr. 2015;101:387–97. 25646337 10.3945/ajcn.114.088617

[15] Nanto-Salonen K , Kupila A, Simell S, Siljander H, Salonsaari T, Hekkala A, Korhonen S, Erkkola R, Sipilä JI, Haavisto L et al. Nasal insulin to prevent type 1 diabetes in children with HLA genotypes and autoantibodies conferring increased risk of disease: a double-blind, randomised controlled trial. Lancet North Am Ed. 2008;372:1746–55. 10.1016/S0140-6736(08)61309-4 18814906

[16] de Boer P , Rahaoui H, Leer RJ, Montijn RC, van der Vossen JM Real-time PCR detection of *Campylobacter* spp.: a comparison to classic culturing and enrichment. Food Microbiol. 2015;51:96–100. 26187833 10.1016/j.fm.2015.05.006

[17] Vu DT , Sethabutr O, Von Seidlein L, Tran VT, Do GC, Bui TC, Le HT, Lee H, Houng HS, Hale TL et al. Detection of *Shigella* by a PCR assay targeting the ipaH gene suggests increased prevalence of shigellosis in Nha Trang, Vietnam. J Clin Microbiol. 2004;42:2031–5. 15131166 10.1128/JCM.42.5.2031-2035.2004 PMC404673

[18] Nurminen N , Juuti R, Oikarinen S, Fan YM, Lehto KM, Maleta CM, Ashorn P, Hyöty H High-throughput multiplex quantitative polymerase chain reaction method for *Giardia lamblia* and *Cryptosporidium* species detection in stool samples. Am J Trop Med Hyg. 2015;92:1222–6. 25918202 10.4269/ajtmh.15-0054 PMC4458829

[19] Krogvold L , Edwin B, Buanes T, Frisk G, Skog O, Anagandula M, Korsgren O, Undlien D, Eike MC, Richardson SJ et al. Detection of a low-grade enteroviral infection in the islets of Langerhans of living patients newly diagnosed with type 1 diabetes. Diabetes. 2015;64:1682–7. 25422108 10.2337/db14-1370

[20] Honkanen H , Oikarinen S, Peltonen P, Simell O, Ilonen J, Veijola R, Knip M, Hyöty H Human rhinoviruses including group C are common in stool samples of young Finnish children. J Clin Virol. 2013;56:250–4. 23273491 10.1016/j.jcv.2012.11.020

[21] Blanton LV , Charbonneau MR, Salih T, Barratt MJ, Venkatesh S, Ilkaveya O, Subramanian S, Manary MJ, Trehan I, Jorgensen JM et al. Gut bacteria that prevent growth impairments transmitted by microbiota from malnourished children. Science. 2016;351:aad3311. 26912898 10.1126/science.aad3311 PMC4787260

[22] Subramanian S , Huq S, Yatsunenko T, Haque R, Mahfuz M, Alam MA, Benezra A, DeStefano J, Meier MF, Muegge BD et al. Persistent gut microbiota immaturity in malnourished Bangladeshi children. Nature. 2014;510:417–21. 24896187 10.1038/nature13421 PMC4189846

[23] WHO Multicentre Growth Reference Study Group WHO child growth standards based on length/height, weight and age. Acta Paediatr Suppl. 2006;450:76–85. 16817681 10.1111/j.1651-2227.2006.tb02378.x

[24] Dewey KG , Hawck MG, Brown KH, Lartey A, Cohen RJ, Peerson JM Infant weight-for-length is positively associated with subsequent linear growth across four different populations. Matern Child Nutr. 2005;1:11–20. 16881875 10.1111/j.1740-8709.2004.00004.x PMC6874388

[25] Bzikowska-Jura A , Czerwonogrodzka-Senczyna A, Olędzka G, Szostak-Węgierek D, Weker H, Wesołowska A Maternal nutrition and body composition during breastfeeding: association with human milk composition. Nutrients. 2018;10:1379. 10.3390/nu10101379 PMC6213543 30262786

[26] Harrela M , Koistinen H, Kaprio J, Lehtovirta M, Tuomilehto J, Eriksson J, Toivanen L, Koskenvuo M, Leinonen P, Koistinen R et al. Genetic and environmental components of interindividual variation in circulating levels of IGF-I, IGF-II, IGFBP-1, and IGFBP-3. J Clin Invest. 1996;98:2612–5. 8958225 10.1172/JCI119081 PMC507720

[27] Kao PC , Matheny AP Jr, Lang CA Insulin-like growth factor-I comparisons in healthy twin children. J Clin Endocrinol Metab. 1994;78:310–2. 8106617 10.1210/jcem.78.2.8106617

[28] Hawkes CP , Grimberg A Insulin-like growth factor-I is a marker for the nutritional state. Pediatr Endocrinol Rev. 2015;13:499–511. 26841638 PMC5576178

[29] Tabecka-Lonczynska A , Mytych J, Solek P, Kowalewski MP, Koziorowski M Seasonal expression of insulin-like growth factor 1 (IGF-1), its receptor IGF-1R and klotho in testis and epididymis of the European bison (*Bison bonasus*, Linnaeus 1758). Theriogenology. 2019;126:199–205. 30579142 10.1016/j.theriogenology.2018.12.016

[30] Perez-Sanchez J , Marti-Palanca H, Le Bail PY Seasonal changes in circulating growth hormone (GH), hepatic GH-binding and plasma insulin-like growth factor-I immunoreactivity in a marine fish, gilthead sea bream, *Sparus aurata*. Fish Physiol Biochem. 1994;13:199–208. 24198190 10.1007/BF00004358

[31] Bubenik GA , Schams D, White RG, Rowell J, Blake J, Bartos L Seasonal levels of metabolic hormones and substrates in male and female reindeer (*Rangifer tarandus*). Comp Biochem Physiol C Pharmacol Toxicol Endocrinol. 1998;120:307–15. 9827045 10.1016/s0742-8413(98)10010-5

[32] Cuzick J. A Wilcoxon-type test for trend. Stat Med. 1985;4:87–90. 3992076 10.1002/sim.4780040112

[33] Cheung YB Statistical analysis of human growth and development. Boca Raton (FL): CRC Press; 2014.

[34] DiLalla LF A structural equation modeling overview for medical researchers. J Dev Behav Pediatr. 2008;29:51–4. 18300720 10.1097/DBP.0b013e31815f250c

[35] Yan J , Charles JF Gut microbiota and IGF-1. Calcif Tissue Int. 2018;102:406–14. 29362822 10.1007/s00223-018-0395-3 PMC6132071

[36] Lofqvist C , Andersson E, Gelander L, Rosberg S, Blum WF, Albertsson Wikland K Reference values for IGF-I throughout childhood and adolescence: a model that accounts simultaneously for the effect of gender, age, and puberty. J Clin Endocrinol Metab. 2001;86:5870–6. 11739455 10.1210/jcem.86.12.8117

[37] Yüksel B , Ozbek MN, Mungan NO, Darendeliler F, Budan B, Bideci A, Çetinkaya E, Berberoğlu M, Evliyaoğlu O, Yeşilkaya E et al. Serum IGF-1 and IGFBP-3 levels in healthy children between 0 and 6 years of age. J Clin Res Pediatr Endocrinol. 2011;3:84–8. 21750637 10.4274/jcrpe.v3i2.17 PMC3119446

[38] Michaelsen KF. Effect of protein intake from 6 to 24 months on insulin-like growth factor 1 (IGF-1) levels, body composition, linear growth velocity, and linear growth acceleration: what are the implications for stunting and wasting?. Food Nutr Bull. 2013;34:268–71. 23964408 10.1177/156482651303400224

[39] Bidlingmaier M , Friedrich N, Emeny RT, Spranger J, Wolthers OD, Roswall J, Körner A, Obermayer-Pietsch B, Hübener C, Dahlgren J et al. Reference intervals for insulin-like growth factor-I (IGF-I) from birth to senescence: results from a multicenter study using a new automated chemiluminescence IGF-I immunoassay conforming to recent international recommendations. J Clin Endocrinol Metab. 2014;99:1712–21. 24606072 10.1210/jc.2013-3059

[40] Prendergast AJ , Rukobo S, Chasekwa B, Mutasa K, Ntozini R, Mbuya MN, Jones A, Moulton LH, Stoltzfus RJ, Humphrey JH Stunting is characterized by chronic inflammation in Zimbabwean infants. PLoS One. 2014;9:e86928. 24558364 10.1371/journal.pone.0086928 PMC3928146

[41] Karlberg J On the modelling of human growth. Statist Med. 1987;6:185–92. 10.1002/sim.4780060210 3589247

[42] Low LC , Tam SY, Kwan EY, Tsang AM, Karlberg J Onset of significant GH dependence of serum IGF-I and IGF-binding protein 3 concentrations in early life. Pediatr Res. 2001;50:737–42. 11726733 10.1203/00006450-200112000-00018

[43] Xu X , Wang W, Guo Z, Karlberg J Longitudinal growth during infancy and childhood in children from Shanghai: predictors and consequences of the age at onset of the childhood phase of growth. Pediatr Res. 2002;51:377–85. 11861945 10.1203/00006450-200203000-00018

[44] DeBoer MD , Scharf RJ, Leite AM, Ferrer A, Havt A, Pinkerton R, Lima AA, Guerrant RL Systemic inflammation, growth factors, and linear growth in the setting of infection and malnutrition. Nutrition. 2017;33:248–53. 27712965 10.1016/j.nut.2016.06.013 PMC5193489

[45] Ameri P , Giusti A, Boschetti M, Bovio M, Teti C, Leoncini G, Ferone D, Murialdo G, Minuto F Vitamin D increases circulating IGF1 in adults: potential implication for the treatment of GH deficiency. Eur J Endocrinol. 2013;169:767–72. 24005315 10.1530/EJE-13-0510

[46] Black RE , Brown KH, Becker S Effects of diarrhea associated with specific enteropathogens on the growth of children in rural Bangladesh. Pediatrics. 1984;73:799–805. 6374599

[47] Guerrant RL , Kirchhoff LV, Shields DS, Nations MK, Leslie J, de Sousa MA, Araujo JG, Correia LL, Sauer KT, McClelland KE Prospective study of diarrheal illnesses in northeastern Brazil: patterns of disease, nutritional impact, etiologies, and risk factors. J Infect Dis. 1983;148:986–97. 6361176 10.1093/infdis/148.6.986

[48] Lima AA , Moore SR, Barboza MS Jr, Soares AM, Schleupner MA, Newman RD, Sears CL, Nataro JP, Fedorko DP, Wuhib T et al. Persistent diarrhea signals a critical period of increased diarrhea burdens and nutritional shortfalls: a prospective cohort study among children in northeastern Brazil. J Infect Dis. 2000;181:1643–51. 10823764 10.1086/315423

[49] Moore SR , Lima AA, Conaway MR, Schorling JB, Soares AM, Guerrant RL Early childhood diarrhoea and helminthiases associate with long-term linear growth faltering. Int J Epidemiol. 2001;30:1457–64. 11821364 10.1093/ije/30.6.1457

[50] Brown KH Diarrhea and malnutrition. J Nutr. 2003;133:328S–32S. 12514320 10.1093/jn/133.1.328S

[51] Stephensen CB Burden of infection on growth failure. J Nutr. 1999;129:534S–8S. 10064326 10.1093/jn/129.2.534S

[52] Hunninghake GW , Doerschug KC, Nymon AB, Schmidt GA, Meyerholz DK, Ashare A Insulin-like growth factor-1 levels contribute to the development of bacterial translocation in sepsis. Am J Respir Crit Care Med. 2010;182:517–25. 20413631 10.1164/rccm.200911-1757OC PMC2937242

[53] Rodrigues LS , Hacker MA, Illarramendi X, Pinheiro MF, Nery JA, Sarno EN, Pessolani MCV Circulating levels of insulin-like growth factor-I (IGF-I) correlate with disease status in leprosy. BMC Infect Dis. 2011;11:339. 22166091 10.1186/1471-2334-11-339 PMC3266221

[54] Helaly GF , El-Afandy NM. Influence of HCV infection on insulin-like growth factor 1 and proinflammatory cytokines: association with risk for growth hormone resistance development. Egypt J Immunol. 2009;16:115–24. 22059359

[55] Prendergast AJ , Humphrey JH. The stunting syndrome in developing countries. Paediatr Int Child Health. 2014;34:250–65. 25310000 10.1179/2046905514Y.0000000158 PMC4232245

[56] Jaidane H , Caloone D, Lobert PE, Sane F, Dardenne O, Naquet P, Gharbi J, Aouni M, Geenen V, Hober D Persistent infection of thymic epithelial cells with coxsackievirus B4 results in decreased expression of type 2 insulin-like growth factor. J Virol. 2012;86:11151–62. 22855493 10.1128/JVI.00726-12 PMC3457166

[57] Maleta K , Virtanen SM, Espo M, Kulmala T, Ashorn P Seasonality of growth and the relationship between weight and height gain in children under three years of age in rural Malawi. Acta Paediatr. 2007;92:491–7. 10.1111/j.1651-2227.2003.tb00584.x 12801119

[58] Waterlow JC. Relationship of gain in height to gain in weight. Eur J Clin Nutr. 1994;48(Suppl 1):S72–4. 8005093

[59] Walker SP , Golden MH. Growth in length of children recovering from severe malnutrition. Eur J Clin Nutr. 1988;42:395–404. 3135181

