# Supplementary material for: Infections and systemic inflammation are associated with lower plasma concentration of insulin-like growth factor I among Malawian children
Source: Am J Clin Nutr. 2020 Dec 31;113(2):380–90. doi: 10.1093/ajcn/nqaa327 (PMC7851819; doi:10.1093/ajcn/nqaa327)
Supplement: nqaa327_Supplemental_File [file nqaa327_supplemental_file.docx]

**Supplementary Figures and Supplementary Table 1**

**Legends of Supplementary Figures:**

**Supplementary Figure 1.** Concept map of the pathways contributing to plasma IGF-1 concentration. Conceptual model of how maternal characteristics and environmental exposures, could contribute to plasma IGF-1 concentration.

Lines in red indicate negative hypothesized associations, lines in blue indicate positive hypothesized associations. Dashed lines from shaded boxes indicate associations that are likely to exist but could not be tested in the current study due to the lack of data on these variables.

AGP, alpha-1-acid glycoprotein; CRP, C-reactive protein; IGF-1, insulin-like growth factor 1; WLZ, weight-for-length Z-score

**Supplementary Figure 2.** Flow diagram of Malawian participants throughout the study.

**Supplementary Figure 3.** Random forest modelling of plasma IGF-1 concentration in Malawian children. In random forest modelling, the plasma IGF-1 concentration was most strongly predicted by systemic inflammation, followed by sex, weight-for-length Z-score, malaria infection, and MAZ.

With random forest modelling, we tested the robustness of identified predictors in the pathway model by determining the importance of the explanatory variables with the change in Mean Squared Error (MSE).

AGP, alpha-1-acid glycoprotein; IGF-1, insulin-like growth factor 1; MAZ, microbiota-for-age Z-score

**Supplementary Figure 1.**

**Supplementary Figure 2.**

**
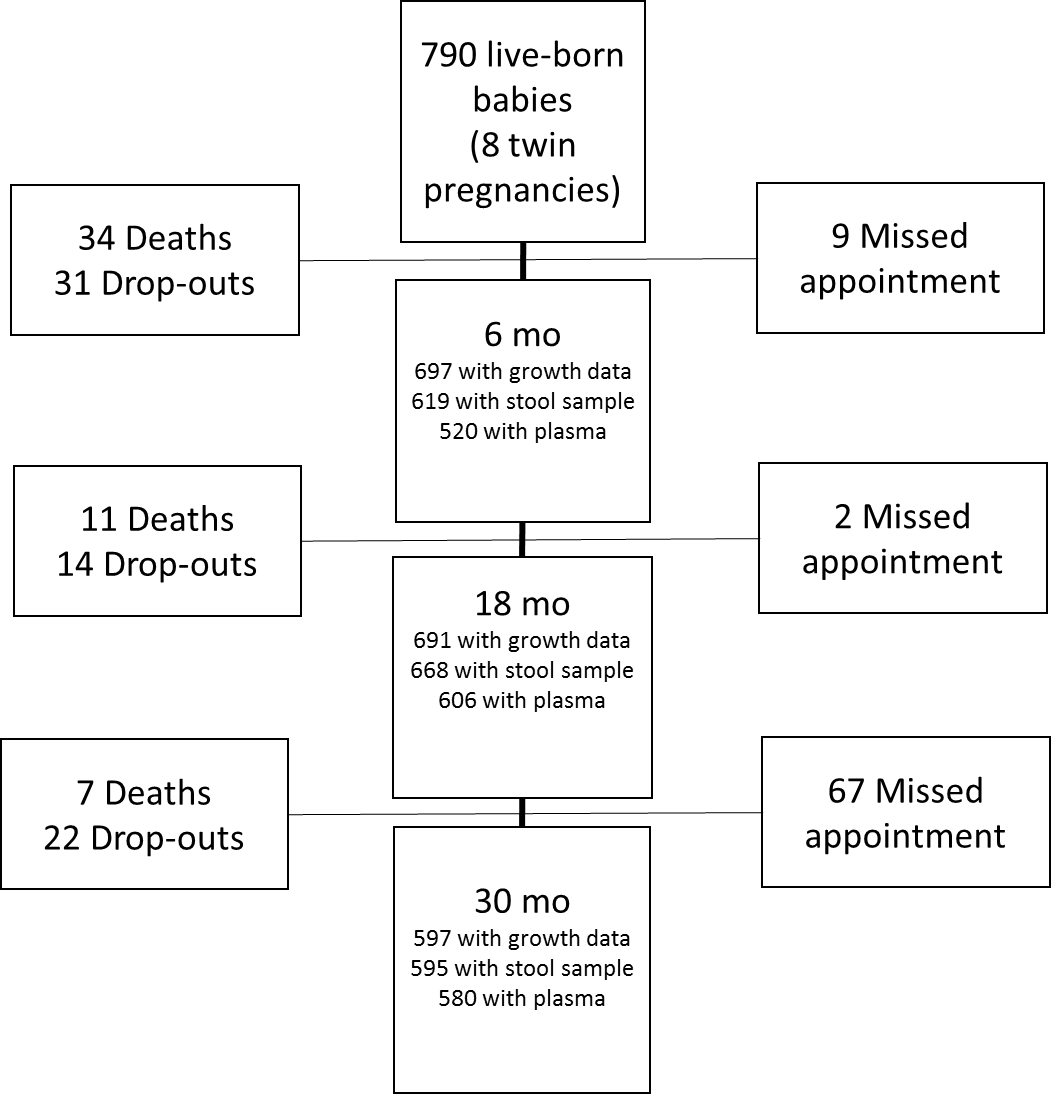
**

**Supplementary Figure 3.**

## **Supplementary Table 1. The association between environmental exposures, maternal characteristics and plasma IGF-1 concentration in Malawian children**

|  | **Mean ± SD plasma IGF-1 concentration, ng/ml** | | | |
| --- | --- | --- | --- | --- |
| **Exposure, risk factor (number of children without exposure/with exposure)** | **Group without exposure** | **Risk group** | **Difference (95% CI)** | **P-value ^7^** |
| Unsafe water source^1^ (552/53) | 12.6 ± 7.8 | 11.4 ± 6.0 | -1.2 (-3.4, 1.0) | 0.28 |
| Unsafe sanitation^2^ (61/544) | 14.9 ± 10.3 | 12.2 ± 7.3 | -2.7 (-5.3, 0.04) | 0.053 |
| Relative poverty in household^3^ (290/315) | 12.7 ± 7.7 | 12.4 ± 7.7 | -0.3 (-1.6, 0.9) | 0.62 |
| Short maternal stature^4^ (528/77) | 12.7 ± 7.7 | 11.2 ± 7.8 | -1.5 (-3.4, 0.3) | 0.10 |
| Maternal malnourishment^5^ (537/68) | 12.4 ± 8.4 | 13.5 ± 7.6 | 1.1 (-0.9, 3.0) | 0.27 |
| Mother has not attended secondary school^6^ (54/513) | 15.2 ± 10.6 | 12.3 ± 7.3 | -3.0 (-5.9, -0.01) | 0.049^8^ |

^1^Unprotected well, lake, or pond

^2^Regular pit latrine or no latrine

^3^Household asset Z-score below median

^4^Maternal height < 150 cm

^5^Maternal BMI < 18.5 kg/m^2^

^6^Mother completed no more than 8 years in school

^7^P-value obtained using Student’s *t*-test

^8^Degrees of freedom for the *t*-test obtained from Welch’s formula due to unequal variances
